# Activating *FGFR1* Mutations in Sporadic Pheochromocytomas

**DOI:** 10.1007/s00268-017-4320-0

**Published:** 2017-11-20

**Authors:** Jenny Welander, Małgorzata Łysiak, Michael Brauckhoff, Laurent Brunaud, Peter Söderkvist, Oliver Gimm

**Affiliations:** 1 Department of Clinical and Experimental Medicine, Faculty of Medicine and Health Sciences, Linköping University, 58185 Linköping, Sweden; 2 Department of Surgery, Haukeland University Hospital, 5021 Bergen, Norway; 3 Department of Clinical Science, University of Bergen, 5020 Bergen, Norway; 4 Department of Digestive, Hepato-Biliary and Endocrine Surgery, CHU Nancy - Hospital Brabois Adultes, University de Lorraine, 54511 Vandoeuvre-les-Nancy, France; 5 Department of Surgery, County Council of Östergötland, 58185 Linköping, Sweden

## Abstract

**Introduction:**

Pheochromocytomas are neuroendocrine tumors of the adrenal glands. Up to 40% of the cases are caused by germline mutations in one of at least 15 susceptibility genes, making them the human neoplasms with the highest degree of heritability. Recurrent somatic alterations are found in about 50% of the more common sporadic tumors with *NF1* being the most common mutated gene (20–25%). In many sporadic tumors, however, a genetic explanation is still lacking.

**Materials and methods:**

We investigated the genomic landscape of sporadic pheochromocytomas with whole-exome sequencing of 16 paired tumor and normal DNA samples and extended confirmation analysis in 2 additional cohorts comprising a total of 80 sporadic pheochromocytomas.

**Results:**

We discovered on average 33 non-silent somatic variants per tumor. One of the recurrently mutated genes was *FGFR1*, encoding the fibroblast growth factor receptor 1, which was recently revealed as an oncogene in pediatric brain tumors. Including a subsequent analysis of a larger cohort, activating *FGFR1* mutations were detected in three of 80 sporadic pheochromocytomas (3.8%). Gene expression microarray profiling showed that these tumors clustered with *NF1*-, *RET,*- and *HRAS*-mutated pheochromocytomas, indicating activation of the MAPK and PI3K-AKT signal transduction pathways.

**Conclusion:**

Besides *RET* and *HRAS*, *FGFR1* is only the third protooncogene found to be recurrently mutated in pheochromocytomas. The results advance our biological understanding of pheochromocytoma and suggest that somatic *FGFR1* activation is an important event in a subset of sporadic pheochromocytomas.

**Electronic supplementary material:**

The online version of this article (10.1007/s00268-017-4320-0) contains supplementary material, which is available to authorized users.

## Introduction

Pheochromocytomas (PCCs) are tumors arising from the neural crest-derived cells of the adrenal medulla, and paragangliomas (PGLs) are their extra-adrenal counterparts. They may cause hypertension due to overproduction of catecholamines, with symptoms including frequent episodes of headache, palpitations and sweating, and an increased risk of cardiovascular disease [1]. PCCs and PGLs have a highly diverse genetic background; up to 40% of the cases are caused by germline mutations in one of at least 15 so far identified susceptibility genes, making them the human neoplasms with the highest degree of heritability [1, 2]. More and more is also known regarding alterations in the sporadic tumors. During the past few years, somatic mutations have been revealed in several of the genes known from the hereditary cases, most frequently in *NF1* [3, 4]. *HRAS* was found as the first gene with recurrent somatic mutations that is not associated with hereditary PCCs (6), and more recently, frequent somatic mutations were also discovered in *ATRX* [5, 6]. Initially, gene expression profiling showed that PCCs could be divided into at least two different clusters based on their expression signature [7]. The first cluster contains tumors with *VHL*, *SDHx,* and *EPAS1* mutations and displays mRNA expression associated with the hypoxic response. The second cluster contains tumors with *RET*, *NF1*, *TMEM127,* and *MAX* mutations and is enriched for mRNA expression related to activation of kinase signaling cascades. Recently, third and fourth clusters have been described [8]. The third cluster contains tumors with somatic *MAML3* fusion genes, *CSDE1* and *ATRX* mutations leading to activation of the Wnt signaling. The fourth cluster contains genes known to be adrenal cortex markers (*CYP11B2*, *CYP21A2*, and *STAR*) and is associated with the presence of cortical cells and termed cortical admixture. Despite this remarkable progress in the field, a large portion of the sporadic tumors still remains without any known genetic driver event [9, 10]. We therefore hypothesized that there are additional targets of recurrent somatic mutations in PCCs. To examine this, we performed whole-exome sequencing of paired tumor and normal DNA from patients with sporadic PCC and followed up the results by studying whole-transcriptome gene expression.

## Materials and methods

### Patients and samples

The discovery cohort of this study consisted of 16 PCCs with corresponding peripheral blood samples from 16 patients operated at Linköping University Hospital, Sweden (Table S1). Findings were further investigated in 15 remaining PCCs from Sweden and Norway with known mutational status in PCC-associated genes (Table S1), followed by 49 additional sporadic PCCs from Hôpital de Brabois, Nancy, France (Table S2). All patients had been diagnosed with sporadic PCC due to absence of family history and syndromic presentation. Two sporadic PCCs and ten tumors from patients with hereditary syndromes were also available and tested (Table S2). Informed consent was obtained from all participants, and the local ethic committees approved the study.

## Methods

All methods are described in the supplementary material.

## Results

### The mutation landscape of pheochromocytomas

We performed whole-exome sequencing of 16 tumors with paired constitutional DNA from patients with apparently sporadic disease (Table S1). Six of the cases had previously tested negative for mutations in known PCC-related genes [4, 9]. Sequencing generated an average depth of 119× for tumor samples and 97× for blood samples, with a depth of at least 10× in 97.9 and 97.5% of the exome, respectively. When investigating somatic mutations in the previously known genes, we detected truncating *NF1* mutations in three of the tumors with previously unknown mutation status, as could be expected in the light of earlier studies [3, 4]. In addition, somatic mutations in the previously known genes *VHL*, *RET*, *MAX,* and *HRAS* were found in one tumor each (Table S3), in agreement with frequencies in previous reports (Table 1) [7, 10–13]. Despite the premises, we did not find any *ATRX* mutations [5, 6]. Moreover, we investigated germline alterations and detected a germline frameshift mutation in *SDHB* in one case for which the mutation status was previously unknown (Table S3). Germline missense variants were also found in *EGLN2*, *KIF1Bβ*, *NF1,* and *SDHD*, but bioinformatic analysis suggests that these are either benign or of unknown significance for the disease (Table S3).

**Table 1:** Frequency of somatic mutations in pheochromocytomas

Gene	*NF1* (%)	*KIF1Bβ* (%)	*ATRX* (%)	*HRAS* (%)	*VHL* (%)	*RET* (%)	*EPAS1* (%)	*MAML3* (%)	*CSDE1* (%)	*MAX* (%)	*FGFR1* (%)
Literature	9–48 [3, 4, 8, 41]	1.5–29.6 [10, 41]	5–12.6 [5, 6]	6.5–10 [8, 13, 42, 43]	1.4–7.9 [7, 8, 10, 42]	2.2–5.6 [7, 8, 10, 42]	2.2–5 [8, 10, 42]	5 [8]	2 [8]	1.5 [10]	0.5 [32]
Present study	18.8	0	0	6.3	6.3	6.3	0	n.d	n.d	3.8	6.3

*n.d* not determined

We detected totally 542 non-silent somatic variants in the cohort of 16 tumors. The median number per tumor was 33 non-silent somatic variants, with a range from 16 to 60 per tumor. Most of the detected alterations were unique to one tumor, but some genes harbored non-silent somatic mutations in two or more samples, including the *NF1* gene as could be expected. The recurrently altered genes also included *FGFR1*, in which activating point mutations were recently discovered in pediatric pilocytic astrocytoma [14].

### Recurrent hotspot *FGFR1* mutations

**Fig. 1:**
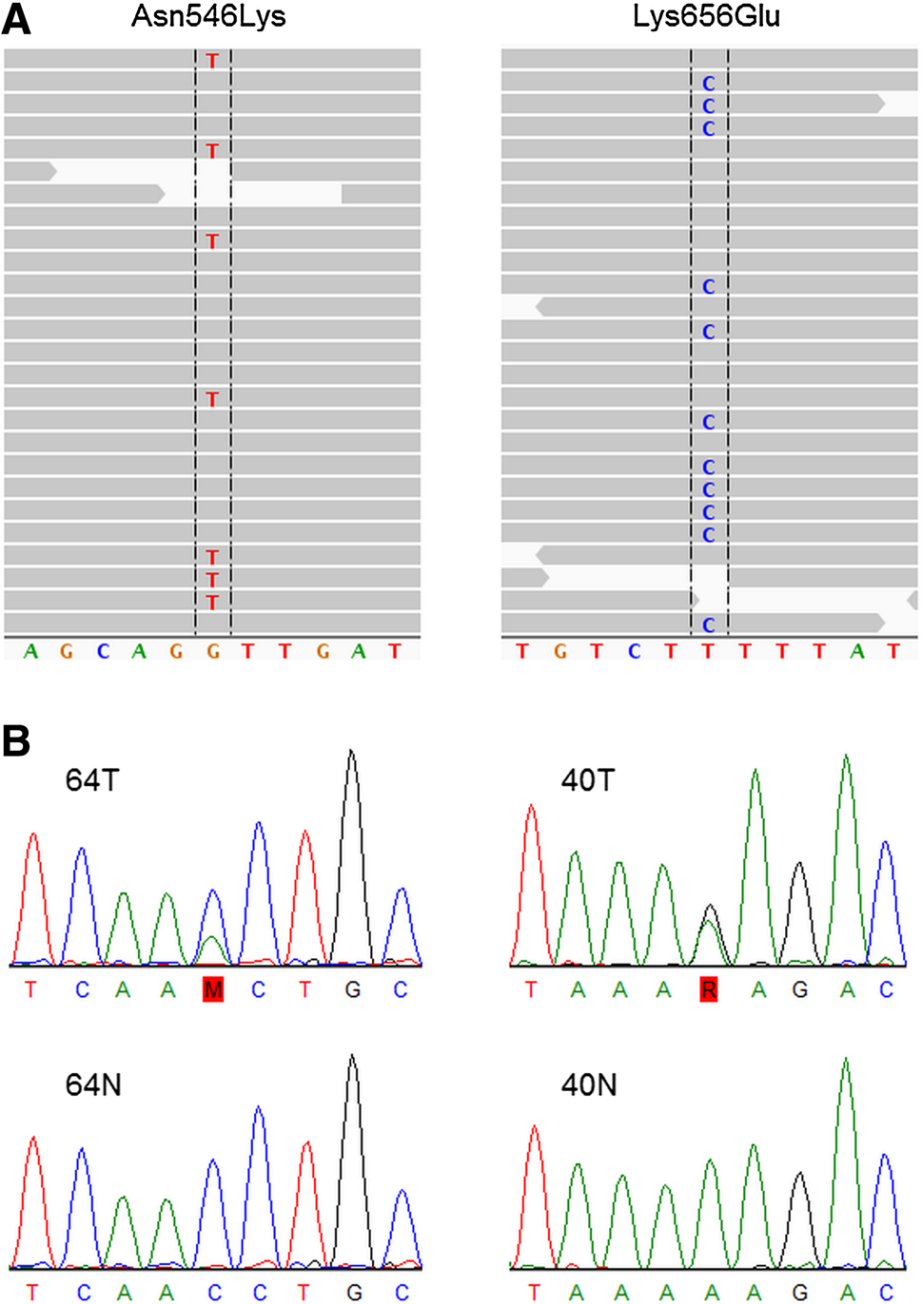
Somatic hotspot mutations in *FGFR1*. **a** Overview of next-generation sequencing reads from the mutated sites in Integrative Genomics Viewer. Read bases that match the hg19 reference are displayed in gray, and mismatches are indicated with color coded alternate alleles (*FGFR1* is oriented on the reverse strand; hence, the sequence is here the reverse complement to the transcribed sequence). **b** Validation of the mutations in the two tumor samples (64T and 40T) with Sanger sequencing (in the direction of transcription) and the corresponding sequences from blood samples

**Fig. 2:**
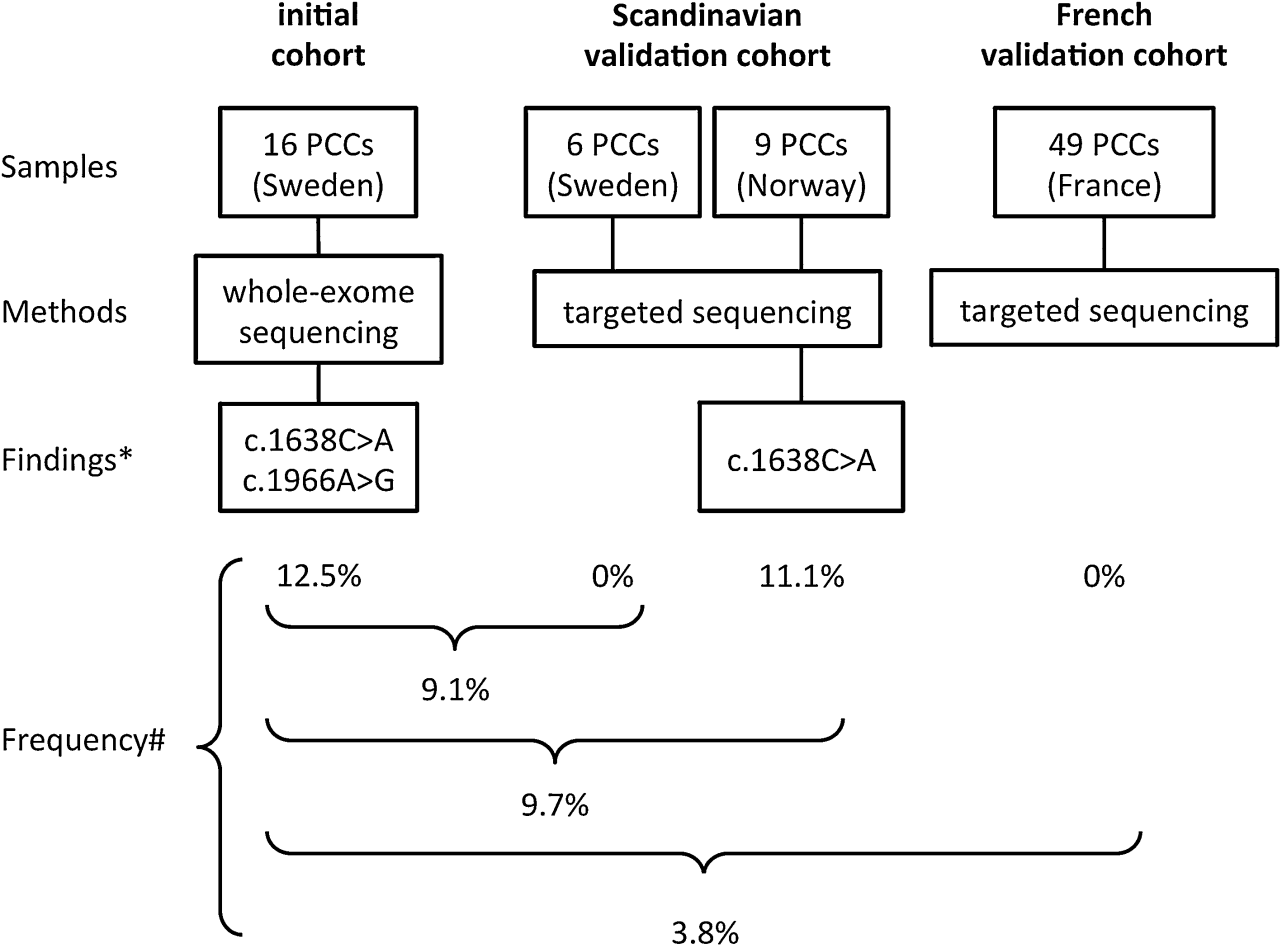
Frequency of *FGFR1* mutations indifferent cohorts. Flow diagram simplifying the process of initial discovery and validation of somatic *FGFR1* mutations in sporadic pheochromocytomas (PCCs). * With regard to somatic *FGFR1* mutations; # of somatic *FGFR1* mutations

We detected two somatic *FGFR1* mutations, Asn546Lys (c.1638C>A) and Lys656Glu (c.1966A>G) (Figs. 1 and 2). They exactly correspond to the two hotspot sites for activating mutations recently discovered in pediatric brain tumors [14]. To investigate the prevalence of *FGFR1* mutations in sporadic PCCs, we performed Sanger sequencing of the three most common hotspots in *FGFR1* (Fig. S1) in 15 remaining tumors from the Scandinavian cohort (Table S1) and detected one additional Asn546Lys mutation (Fig. 2). Subsequent analysis of 49 sporadic PCCs from France (Table S2) did not reveal any additional mutations (Fig. 2). In total, the combined cohort consisted of 80 apparently sporadic PCCs, of which three tumors (samples #40, #50, #64; 3.8%) carried *FGFR1* mutations (Fig. 2). The cases with somatic *FGFR1* mutations were two females and one male with a mean age of 66 years (Table 2). One of the *FGFR1*-mutated tumors also had a somatic *MAX* mutation, whereas the other two did not have any mutations in known PCC-associated genes (Fig. S2). Apart from the main cohort of sporadic PCCs, we tested also two sporadic PGLs and ten hereditary PCCs (four MEN2B, two VHL, three PGL1, and one PGL4), yet no *FGFR1* mutations were detected. In addition, we searched samples with available RNA (Table S4) for a known fusion gene involving *FGFR1*, i.e., *FGFR1*-*TACC1* [15, 16], which is known to play an oncogenic role in glioblastomas, but no gene fusions were found in PCCs.

**Table 2:** Clinical and genetic data for cases with somatic *FGFR1* mutations

Case ID	Gender	Age (years)	Tumor size (mm)	Malignancy	Mutation^a^	Protein alteration	Copy number
40	Female	63	32	Benign	c.1638C>A	Asn546Lys	Gain
50	Female	80	10	Benign	c.1966A>G	Lys656Glu	Normal
64	Male	56	32	Benign	c.1638C>A	Asn546Lys	Normal

^a^Mutations were annotated according to the Ensembl transcript ENST00000447712

### Copy number gains at the *FGFR1* locus

Copy number analysis showed a gain of *FGFR1* in four of 80 sporadic PCCs, one of which also had a *FGFR1* mutation (Fig. S3). SNP microarray data from a previous study [4] were used to verify these findings where possible. For the sample with both *FGFR1* copy gain and *FGFR1* mutation, SNP microarray data were available and amplification of the *FGFR1* locus was confirmed and microarray data showed that it was part of a ~2.5 Mb amplification unit on chromosome 8p (Fig. S4). Copy number loss in the *FGFR1* region was seen in 11/91 sporadic PCCs (Table S4), none of which carried any *FGFR1* mutations. For three of four cases with loss and where microarray data were also available, the loss could be seen to cover large parts of chromosome 8, suggesting that loss of *FGFR1* in these cases is a passenger event (for the fourth sample, a loss was not detectable using the array technique, possibly because of a too low marker resolution).

### Gene expression profiling of *FGFR1*-mutated tumors

Global gene expression analysis showed that the three PCCs with *FGFR1* mutations clustered in “cluster 2” together with tumors that contained mutations in *RET*, *NF1,* and *HRAS.* This indicates that *FGFR1* mutations are associated with activation of kinase signal transduction pathways, including the MAPK and PI3K-AKT pathways [9]. Among all the genes that passed quality control, there were no genes that differed significantly in expression between tumors with and without *FGFR1* mutations after correction for multiple testing. The reasons for this may be that the *FGFR1*-mutated tumors have a gene expression profile very similar to the other tumors in “cluster 2” and that the number of tested *FGFR1*-mutated tumors was too small to detect minor differences in expression patterns. There was no association between *FGFR1* mutations and *FGFR1* gene expression, as would be expected since the mutations are thought to have their effect on the protein level. However, copy gain of *FGFR1* was associated with an enhanced *FGFR1* gene expression (Fig. S5), suggesting a gene-dose effect.

### Analysis of other fibroblast growth factor receptors

To further extend the knowledge of fibroblast growth factor receptors in PCCs, exons containing hotspot regions in *FGFR2* and *FGFR3* (Fig. S1) were investigated for mutations in all samples (sporadic and hereditary), but no mutations were detected. A very rare polymorphism in *FGFR3* (rs17881656, Phe384Leu) was detected in three cases (two of which were hereditary) and was also present in normal DNA of the two patients where blood was available (Table S4). The variant was predicted as benign using the Polyphen-2, SIFT, and PROVEAN algorithms [17–19]. Allele frequency was then analyzed in a regional healthy control population (*n* = 739), as well as checked in the Swedish 1000 Genomes population (*n* = 1000; https://swegen-exac.nbis.se/) and the ExAC database (http://exac.broadinstitute.org/). The C-allele frequency was 1.6% in the PCC-cohort and 0.34, 0.4 and 0.31% in the regional control cohort, the Swedish 1000 Genome database and the ExAC database, respectively. Two-tailed Fisher’s exact test showed a borderline significant difference when compared to the Swedish control population (*p* = 0.049), the SweGen database (*p* = 0.07), and the ExAC database (*p* = 0.02). A rare germline variant in *FGFR1* was detected in one case (c. 381T>G, Asp127Glu, rs750795714) and reported in only 2 out of 120,880 alleles in the ExAC database. It was outside of the mutational hotspots and was predicted as benign by PolyPhen. The known gene fusion involving *FGFR3* (*FGFR3*-*TACC3* [15, 16]) was also investigated, but no gene fusions were discovered. Available DNA microarray data from 21 tumors did not reveal any copy number alterations in any of the *FGFR1*-*3* genes other than *FGFR1*. A summary of *FGFR1*-*3* sequence variations, copy number alterations, and gene expression levels for all samples is given in Table S4.

## Discussion

In this report, we present evidence that activating mutations in a gene encoding for fibroblast growth factor receptor 1, *FGFR1*, are important somatic events in some sporadic PCCs. FGFRs, which in humans include FGFR1 to FGFR4, are receptor tyrosine kinases that are involved in multiple processes during embryonic development, including mesenchymal–epithelial communication and formation of several organ systems [20, 21]. In the adult, fibroblast growth factor receptor signaling is involved in tissue homeostasis and regulates processes such as tissue repair, angiogenesis, and inflammation. Binding of FGFs to FGFRs induces receptor dimerization that activates the intracellular kinase domain and leads to transphosphorylation [21]. The phosphorylated tyrosine residues function as docking sites for adaptor proteins, for example FRS2, which can be phosphorylated by FGFRs. This leads to an activation of several signal transduction pathways, including the RAS-RAF-MAPK and PI3K-AKT pathways. The most established link between FGFRs and cancer is probably with bladder cancer, in which *FGFR3* is one of the most commonly mutated genes [22] and the mutations are associated with a non-invasive behavior [23]. Mutations in *FGFR2* and *FGFR3* have also been reported in other tumor forms, whereas activation of FGFR1 has mainly been observed in the form of *FGFR1* amplification in breast and lung cancer [20]. However, occasional point mutations and fusion genes involving *FGFR1* have been observed in glioblastomas [24], which is in agreement with FGFR1 being the most abundant fibroblast growth factor receptor in the nervous system [25]. Most recently, frequent *FGFR1* mutations in two hotspot sites were revealed in another brain tumor: pilocytic astrocytoma [14]. The two variants detected in this study, Asn546Lys and Lys656Glu, affect the kinase domain and have been shown to promote cell proliferation [26], and Asn546Lys also alter FGFR1 autophosphorylation [27]. Furthermore, overexpression of FGFR1 results in MAPK and AKT activation and neurite outgrowth in the rat pheochromocytoma cell line PC12 [25].

**Fig. 3:**
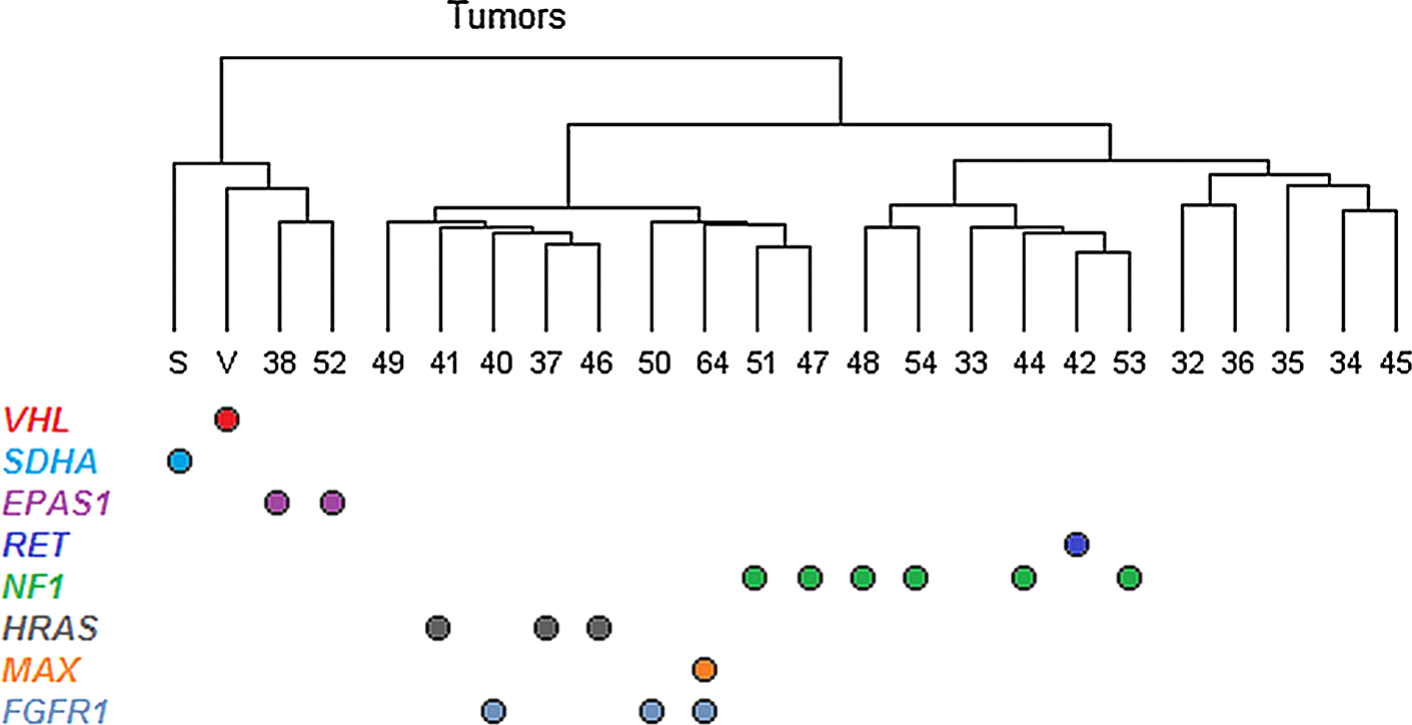
Hierarchical clustering of tumors based on gene expression levels. Mutation status is indicated below the dendrogram, showing that tumors with *FGFR1* mutations cluster together with those that have *RET*, *NF1,* or *HRAS* mutations. Whereas mutations in the known susceptibility genes were mutually exclusive, one mutation in the novel susceptibility gene, *FGFR1*, occurred in combination with a somatic *MAX* mutation

In this study, we discovered that the same *FGFR1* hotspot residues as in certain brain tumors, Asn546 and Lys656 (also reported in 1 of 394 PCCs in the COSMIC database [28]), are recurrently mutated in PCCs. Interestingly, the first cohort investigated in this study, which consisted of Scandinavian cases, had a prevalence of *FGFR1* mutations of almost 10%. However, further investigation in a French cohort did not reveal any additional mutations, resulting in a total frequency of 3.8% (Fig. 2) in the combined cohorts of 80 sporadic PCCs. This means that somatic *FGFR1* mutations are a rare but recurrent event in PCCs (Table 1). The difference in the frequency of somatic *FGFR1* mutations between PCCs from different countries may be due to random variation, or, alternatively, there could be regional differences in the frequency of mutations. Data on the ethnic background are not available in our cohort. Possibly, regional differences in the occurrence of certain germline variants could alter the predisposition to obtain somatic *FGFR1* mutations. Such a phenomenon has previously been observed for a polymorphism that is associated with a hypermutability of the *APC* gene [29] involved in colorectal cancer. The significance of *FGFR1* mutations in PCCs is also supported by data from the TCGA cohort of PCCs and PGLs (http://cancergenome.nih.gov/). Analyzed sequencing data accessed through cBioPortal [30, 31]) displayed a prevalence of Asn546Lys mutation (corresponding to the Asn577Lys in the cBioPortal) in 2/173 (1.2%) of the samples. Interestingly, Toledo et al. very recently found the Asn546Lys mutation in 1/43 PCCs/PGLs, but none in a 136 patient validation cohort supporting the role of FGFR1 mutation in PCCs/PGLs as a rare but recurrent event in three different cohorts at a frequency similar to several other PCC/PGL susceptibility genes [32]. In addition, we observed copy number gains in the *FGFR1* region, which were associated with an increased *FGFR1* gene expression, which is in agreement with an oncogenic role of *FGFR1* in PCCs. Our findings constitute an additional PCC susceptibility gene associated with MAPK and AKT activation, and indeed, gene expression profiling showed that the *FGFR1*-mutated tumors clustered together with tumors having mutations in the *NF1*, *RET,* and *HRAS* genes (Fig. 3). Future studies in larger cohorts will be valuable to more accurately estimate the frequency and the potential clinical consequences of *FGFR1* alterations in PCCs/PGLs. The potential association of the *FGFR3* variant Phe384Leu (rs17881656) with PCCs warrants further examination.

The whole-exome sequencing approach used in this study also detected a median of 33 non-silent somatic mutations in each tumor. Most of the altered genes detected here were only altered in a single sample, suggesting that there is a large diversity of somatic events occurring in PCCs. Many of these may be passenger events, and further studies with larger sample sizes will probably be required in order to identify additional true but infrequent drivers in the tumorigenesis [33]. In agreement with previous reports [34, 35], the exome sequencing technique was also useful in order to find a germline *SDHB* mutation in a patient with apparently sporadic PCCs, but we also detected numerous variants of unknown significance that may be difficult to interpret in a clinical setting. For gene and pathway discovery, however, next-generation sequencing and omics approaches have been incredibly successful [34, 36–38] and will likely result in the detection of additional PCC/PGL-associated genes in the near future. Indeed, recent exome sequencing initiatives published during the finishing of this work have revealed additional genes, including *ATRX*, *MDH2,* and *KMT2D*, to be potentially involved in PCC/PGL pathogenesis [5, 6, 39, 40], and *FGFR1* can now be added to the list of PCC susceptibility genes. This finding advances our biological understanding of PCC development and possibly opens for novel therapeutic options involving FGFR inhibitors, which are currently being evaluated in other tumor forms [21].

## Electronic supplementary material

Below is the link to the electronic supplementary material.

Supplementary material 1 (DOCX 655 kb)
