# Supplementary material for: Activating FGFR1 Mutations in Sporadic Pheochromocytomas
Source: World J Surg. 2017 Nov 20;42(2):482–9. doi: 10.1007/s00268-017-4320-0 (PMC5762800; doi:10.1007/s00268-017-4320-0)
Supplement: Supplementary file 1 — Supplementary material 1 (DOCX 655 kb) [file 268_2017_4320_MOESM1_ESM.docx]

**Activating *FGFR1* mutations in sporadic pheochromocytomas**

Jenny Welander, Małgorzata Łysiak, Michael Brauckhoff, Laurent Brunaud, Peter Söderkvist , Oliver Gimm

**Corresponding author:** Peter Söderkvist, Department of Clinical and Experimental Medicine, Faculty of Medicine and Health Sciences, Linköping University, SE-58185 Linköping, Sweden.

Email: [peter.soderkvist@liu.se](mailto:peter.soderkvist@liu.se)

**Supplementary Material**

**Supplemental materials and methods**

**Preparation of DNA and RNA**

DNA and RNA isolation was performed as previously described [1].

## Whole-exome enrichment, next-generation sequencing and data analysis

Exome capture from tumor and blood genomic DNA was performed using the SureSelect Human All Exon V5 target enrichment kit. Paired-end sequencing (2x100bp) was performed on an Illumina HiSeq 2500 instrument with high output mode after cluster generation using cBot (Illumina). DNA samples were multiplexed with three tumor samples per lane and 4.2 blood samples per lane.

Raw data files were converted to Fastq format using bcl2Fastq v1.8.3 from the CASAVA software suite (Illumina). Sequencing reads were mapped to the human reference genome hg19 using the Burrows-Wheeler Aligner (BWA) v.0.6.2-r126 [2] and sorting and duplicate removal was performed with Picard v. 1.64 (http://broadinstitute.github.io/picard). To study germline mutations, the Genome Analysis Toolkit (GATK) v. 1.5-11-g5c5d8e7 [3] was used to call variants in normal DNA according to the best practice procedure for data cleanup implemented at the BROAD Institute [4]. Next, we used the alignment (bam) files from tumor and corresponding normal DNA for somatic variant calling. Single nucleotide substitutions were called using MuTect v. 1.1.5 [5] with default settings. Insertions and deletions (indels) were called using VarScan v. 2.3.7 [6] according to the instructions of the developer, including conversion of alignment files to the pileup format with SAMtools v. 0.1.19 [7], and only variants classified as somatic and high-confidence by VarScan were used in downstream analysis. Annotation and effect prediction of variants was performed with SnpEff v. 3.6 [8]. We excluded all silent variants based on the SnpEff annotations, resulting in a final list of 542 non-synonymous somatic variants that was searched for recurrently altered genes. The results from germline as well as somatic variant calling from all cases were also searched for mutations in known susceptibility genes [9], also including *EPAS1*/*HIF2A* [10], *FH* [11, 12], *HRAS* [13] and *EGLN2*/*PHD1* [14] which have recently been associated with PCC/PPGL and *IDH1* [15] and *BAP1* [16] which have been suggested in single cases. Mutations were visualized by loading bam files into the Integrative Genomics Viewer [17, 18]. All identified mutations were confirmed by Sanger dideoxy termination sequencing.

## Genotyping

## The prevalence of a polymorphism found in *FGFR3* (rs17881656) was analyzed in a healthy control population of 739 adult individuals randomly collected from south-east Sweden with approximately 1:1 ratio of male to female. The rs17881656 was genotyped using TaqMan SNP Genotyping assay (C_58182643_10) with 20ng of genomic DNA extracted from blood using Maxwell 16 Blood DNA Purification Kit (Promega), mixed with TaqMan Universal PCR Master Mix (2X), assay mix (40X) and water according to the manufacturer’s protocol. Analysis was performed in the ABI Prism 7900 Sequence Detection System using SDS 2.4 software for allelic discrimination (Applied Biosystems).

## Copy number analysis

Copy number data had previously been retrieved with SNP microarray analysis (GeneChip Human Mapping 250K, Affymetrix) for 21 tumors in the Scandinavian cohort [19] and was analyzed for copy number alterations in *FGFR1*, *FGFR2*, *FGFR3* and *FGFR4* using the Genotyping Console Browser v. 4.0 (Affymetrix). In addition, DNA copy number in the *FGFR1* gene was investigated with digital droplet PCR [20] in all samples using the QX100 Droplet Digital PCR system (BioRad) (Table S4). DNA was digested with the *Haelll* restriction enzyme (Thermo Scientific, 1 unit per 100 ng of DNA) for 1 hour in 37 ̊ C. Fifteen ng of digested DNA was mixed with ddPCR Supermix for Probes (BioRad), ddPCR probe assay specific for the *FGFR1* (dHsaCP2500319, BioRad), labeled with FAM dye, ddPCR probe assay specific for the reference gene *AP3B1* (dHsaCP1000001, BioRad) labeled with HEX dye and water according to the manufacturer’s instructions, and the reaction mix was then emulsified into droplets surrounded by oil. Amplification and counting of fluorescent droplets was performed according to the manufacturer’s protocol. Data was analysed using the QuantaSoft software v. 1.2.10 (BioRad) and automated clustering analysis. Assuming that DNA was extracted from samples with at least 50% of tumor cells, a copy number higher than 2.5 was considered to be an amplification and a copy number lower than 1.5 was considered a deletion.

**Microarray-based and targeted gene expression analysis**

Tumor RNA from 21 of the 31 cases in the Scandinavian cohort, including two tumors with *FGFR1* mutations, had previously been analysed with GeneChip Human Gene 1.0 ST arrays (Affymetrix) [1], and the third tumor with an *FGFR1* mutation was now analyzed with the same methodology and added to the dataset. Tumors with known somatic *RET*, *NF1*, *HRAS* and *EPAS1* mutations were present in the dataset, and tumors from two hereditary cases, one with a *VHL* mutation and one with an *SDHA* mutation, were used as controls. Microarray data files were analyzed with GeneSpring GX v. 12.6 (Agilent Technologies). Pre-processing, including background correction and normalization, was performed with the robust multi-array average (RMA) algorithm [21]. A quality filter was applied which removed probe sets for which none of the 24 samples had signal intensity values greater than the 20th percentile of all signal intensity values of the sample, leaving 24 908 of the total 28 869 probe sets for downstream analysis. Genes passing the initial quality control were tested for differential expression between samples with and without *FGFR1* mutations using t-tests with Benjamini-Hochberg correction for multiple testing [22], with an accepted false discovery rate of 0.05. Hierarchical clustering was performed as previously described, with 400 probe sets that remained after the quality filtering of a previously defined gene set observed to separate tumors of different genetic backgrounds [1, 23].

The QX100 Droplet Digital PCR system (BioRad) was used to assess the relative gene expression of *FGFR1* all samples with available RNA (Table S4). cDNA was obtained as previously described [19] and 0.5 ng of RNA-equivalent cDNA was used for each sample. cDNA was mixed with ddPCR Supermix for Probes, ddPCR probe assay specific for *FGFR1* gene expression (dHsaCPE5032990, BioRad) labelled with FAM dye, ddPCR probe assay specific for *GAPDH* chosen to be a reference gene (dHsaCPE5031597, BioRad) labeled with HEX dye and water. Droplet generation, amplification and counting of fluorescent droplets were performed according to the manufacturer’s protocol. The *FGFR1* RNA expression level was duplexed with and normalized to the expression of *GAPDH* (S6 Table). The QuantaSoft software v. 1.2.10 was used to analyze the data. Thresholds were adjusted manually.

The QX100 Droplet Digital PCR system (BioRad) was used to assess the relative gene expression of *FGFR1* all samples with available RNA (Table S4).

## Sanger sequencing

Capillary Sanger sequencing of exons 4, 12 and 14 (containing hotspot mutation sites as shown in Fig. S1) of the *FGFR1* gene was performed as previously described [19] with primers as specified in Table S5 in order to confirm the mutations detected with exome sequencing and to screen additional samples. *FGFR1* mutations were annotated according to the Ensembl transcript ENST00000447712 which corresponds to the canonical isoform in the UniProt database. Hotspot regions in *FGFR2* and *FGFR3* were derived through mutation statistics in the COSMIC database (Fig. S6), and were sequenced using primers specified in Table S6 for all samples that were not already covered by exome sequencing. Also, known oncogenic *FGFR1* and *FGFR3* fusion genes [24], *FGFR1-TACC1* and *FGFR3-TACC3*, were searched for in all samples where RNA was available, using previously published primers [25]. RNA was converted to cDNA as previously described [19].

The germline *SDHB* mutation detected in one case was also confirmed with Sanger sequencing with previously designed primers [19]. To complete the genetic knowledge of the cohort, all Scandinavian tumor samples not included for exome sequencing were also analyzed for *HRAS* mutations (Tables S7 and S8), which were present in four of 31 cases (12.9%).

**Table S1. Cases included in the first part of the study analysed by exome or targeted sequencing.**

The samples are pheochromocytomas from patients with apparently sporadic disease surgically removed in Linköping, Sweden (32-45, 57-68) and in Bergen, Norway (46-54).

| **Case ID** | **Age**  **[years]** | **Gender**  **[male (M) or female (F)]** | **Tumor size [mm]** | | **Malignancy** | | **Mutation**  **[somatic (S) or germline (G)]** | **Analyzed with whole-exome sequencing** | **Analyzed with targeted sequencing^a^** | **Plasma normeta-nephrine^b^** | **Plasma meta-nephrine^b^** |
| --- | --- | --- | --- | --- | --- | --- | --- | --- | --- | --- | --- |
| 32 | 39 | M | 30 | Benign | |  | | Yes | Yes | 8.67 | 0.67 |
| 33 | 76 | F | 40 | Benign | |  | | Yes | Yes | 8.17 | 3.33 |
| 34 | 54 | M | 17 | Benign | |  | | Yes | Yes | 5.33 | 1.00 |
| 35 | 75 | M | 60 | Benign | |  | | Yes | Yes | n.d. | n.d. |
| 40 | 63 | F | 32 | Benign | | *FGFR1* (S) | | Yes | Yes | 2.50 | 24.67 |
| 45 | 58 | M | 30 | Benign | |  | | Yes | Yes | n.d. | n.d. |
| 57 | 58 | M | 30 | Relapse but considered benign | | *NF1* (S) | | Yes | *FGFR1* only | 3.0 | 7.7 |
| 58 | 48 | F | 35 | Benign | |  | | Yes | *FGFR1* only | n.d. | n.d. |
| 60 | 78 | M | 80 | Benign | | *RET* (S) | | Yes | *FGFR1* only | 21.67 | 30.67 |
| 61 | 63 | F | 100 | Liver infiltration, patient died after 7 months | | *NF1* (S) | | Yes | *FGFR1* only | 15.67 | 190.00 |
| 62 | 33 | M | 26 | Benign | | *VHL* (S) | | Yes | *FGFR1* only | 6.33 | 0.67 |
| 63 | 69 | M | 35 | Benign | |  | | Yes | *FGFR1* only | 5.00 | 2.00 |
| 64 | 56 | M | 32 | Benign | | *FGFR1* (S), *MAX* (S) | | Yes | *FGFR1* only | 2.50 | 6.33 |
| 66 | 26 | F | 50 | Benign | | *NF1* (S) | | Yes | *FGFR1* only | 13.00 | 29.33 |
| 67 | 38 | M | 55 | Benign | | *SDHB* (G) | | Yes | *FGFR1* only | 7.50 | 1.00 |
| 68 | 66 | F | 50 | Benign | | *HRAS* (S) | | Yes | *FGFR1* only |  |  |
| 36 | 71 | F | 35 | Benign | |  | | No | Yes | 4.83 | 8.33 |
| 37 | 70 | F | 30 | Benign | | *HRAS* (S) | | No | Yes | 3.17 | 3.67 |
| 38 | 47 | M | 30 | Benign | | *EPAS* (G and S) | | No | Yes | 10.50 | 1.00 |
| 41 | 68 | F | 20 | Benign | | *HRAS* (S) | | No | Yes | n.d. | n.d. |
| 42 | 62 | F | 30 | Benign | | *RET* (S) | | No | Yes | n.d. | n.d. |
| 44 | 68 | F | 55 | Benign | | *NF1* (S) | | No | Yes | n.d. | n.d. |
| 46 | 54 | F | 50 | Benign | | *HRAS* (S) | | No | Yes | 1.77 | 27.39 |
| 47 | 42 | M | 90 | Benign | | *NF1* (S) | | No | Yes | 19.61 | 40.11 |
| 48 | 59 | F | 60 | Benign | | *NF1* (S) | | No | Yes | 20.09 | 19.85 |
| 49 | 76 | F | 25 | Benign | |  | | No | Yes | 3.16 | 4.91 |
| 50 | 80 | F | 10 | Benign | | *FGFR1* (n.d.)^c^ | | No | Yes | 1.98 | 1.54 |
| 51 | 43 | M | 55 | Benign | | *NF1* (S) | | No | Yes | 4.58 | 22.61 |
| 52 | 43 | F | 37 | Benign | | *EPAS1* (S) | | No | Yes | 6.63 | 0.72 |
| 53 | 63 | F | 80 | Benign | | *NF1* (S) | | No | Yes | 8.16 | 26.96 |
| 54 | 58 | M | 20 | Benign | | *NF1* (S) | | No | Yes | 13.30 | 2.70 |

S, Somatic; G, Germline; F, Female; M, male; n.d., no data.

^a^Analyzed with Sanger sequencing for *RET*, *VHL*, *NF1*, *SDHB*, *SDHD*, *MAX*, *TMEM127*, *EPAS1* (as previously published(1, 2)), *HRAS* (Table S7 and S8) and *FGFR1* (Table 1 in main paper).

^b^Plasma metanephrines levels have been normalized to the value considered normal for the measuring method. Values within the normal reference range are ≤1.

^c^Blood DNA was not available.

**Table S2. Pheochromocytomas and paragangliomas from Nancy, France, used for additional mutation analysis in *FGFR1*.**

The cohort included 49 sporadic pheochromocytomas, two sporadic paragangliomas (F27, F43) and ten hereditary tumors (F2, F4, F6, F7, F11, F24,F30, F47, F60 and F61).

| **Case ID** | **Age**  **[years]** | **Gender** | **Tumor size [mm]** | **Malignancy** | **Syndrome/known germline mutation** | **Tumor** |
| --- | --- | --- | --- | --- | --- | --- |
| F1 | 24 | F | 40 | Benign | Sporadic | Pheochromocytoma |
| F3 | 78 | M | 70 | Benign | Sporadic | Pheochromocytoma |
| F5 | 57 | F | 30 | Benign | Sporadic | Pheochromocytoma |
| F8 | 60 | M | 35 | Benign | Sporadic | Pheochromocytoma |
| F9 | 37 | F | 100 | Benign | Sporadic | Pheochromocytoma |
| F10 | 70 | M | 40 | Benign | Sporadic | Pheochromocytoma |
| F12 | 30 | M | 35 | Benign | Sporadic | Pheochromocytoma |
| F14 | 70 | F | 37 | Benign | Sporadic | Pheochromocytoma |
| F15 | 52 | F | 20 | Benign | Sporadic | Pheochromocytoma |
| F16 | 75 | F | 70 | Benign | Sporadic | Pheochromocytoma |
| F17 | 40 | F | 40 | Benign | Sporadic | Pheochromocytoma |
| F18 | 57 | M | 25 | Benign | Sporadic | Pheochromocytoma |
| F19 | 66 | M | 27 | Benign | Sporadic | Pheochromocytoma |
| F20 | 46 | M | 37 | Benign | Sporadic | Pheochromocytoma |
| F21 | 83 | M | 50 | Benign | Sporadic | Pheochromocytoma |
| F22 | 63 | M | 80 | Benign | Sporadic | Pheochromocytoma |
| F23 | 34 | M | 20 | Benign | Sporadic | Pheochromocytoma |
| F25 | 47 | F | 40 | Benign | Sporadic | Pheochromocytoma |
| F26 | 62 | F | 50 | Benign | Sporadic | Pheochromocytoma |
| F27 | 61 | M | 27 | Benign | Sporadic | Paraganglioma |
| F28 | 64 | M | 55 | Benign | Sporadic | Pheochromocytoma |
| F29 | 83 | M | 50 | Benign | Sporadic | Pheochromocytoma |
| F31 | 48 | F | 60 | Benign | Sporadic | Pheochromocytoma |
| F32 | 64 | F | 47 | Benign | Sporadic | Pheochromocytoma |
| F33 | 66 | M | 21 | Benign | Sporadic | Pheochromocytoma |
| F34 | 59 | M | 30 | Benign | Sporadic | Pheochromocytoma |
| F35 | 80 | M | 90 | Benign | Sporadic | Pheochromocytoma |
| F36 | 40 | M | 30 | Benign | Sporadic | Pheochromocytoma |
| F37 | 66 | F | 40 | Benign | Sporadic | Pheochromocytoma |
| F38 | 69 | M | 30 | Benign | Sporadic | Pheochromocytoma |
| F39 | 55 | F | 90 | Benign | Sporadic | Pheochromocytoma |
| F40 | 44 | F | 30 | Benign | Sporadic | Pheochromocytoma |
| F41 | 58 | F | 50 | Benign | Sporadic | Pheochromocytoma |
| F42 | 62 | F | 50 | Benign | Sporadic | Pheochromocytoma |
| F43 | 51 | F | 30 | Benign | Sporadic | Paraganglioma |
| F44 | 41 | M | 33 | Benign | Sporadic | Pheochromocytoma |
| F45 | 45 | F | 38 | Benign | Sporadic | Pheochromocytoma |
| F46 | 27 | F | 40 | Benign | Sporadic | Pheochromocytoma |
| F48 | 49 | F | 40 | Benign | Sporadic | Pheochromocytoma |
| F49 | 67 | F | 50 | Benign | Sporadic | Pheochromocytoma |
| F50 | 48 | F | 60 | Benign | Sporadic | Pheochromocytoma |
| F51 | 52 | F | 25 | Benign | Sporadic | Pheochromocytoma |
| F52 | 36 | F | 45 | Benign | Sporadic | Pheochromocytoma |
| F53 | 50 | F | 25 | Benign | Sporadic | Pheochromocytoma |
| F54 | 52 | F | 50 | Benign | Sporadic | Pheochromocytoma |
| **Case ID** | **Age**  **[years]** | **Gender** | **Tumor size [mm]** | **Malignancy** | **Syndrome/known germline mutation** | **Tumor** |
| F55 | 72 | M | 40 | Benign | Sporadic | Pheochromocytoma |
| F56 | 30 | M | 65 | Benign | Sporadic | Pheochromocytoma |
| F57 | 63 | M | 40 | Benign | Sporadic | Pheochromocytoma |
| F58 | 44 | M | 50 | Benign | Sporadic | Pheochromocytoma |
| F59 | 61 | F | 45 | Benign | Sporadic | Pheochromocytoma |
| F62 | 84 | F | 30 | Benign | Sporadic | Pheochromocytoma |
| F2 | 30 | F | >100 | Malignant | PGL4/*SDHB* | Pheochromocytoma |
| F4 | 29 | M | 10 | Benign | MEN2/*RET* | Pheochromocytoma |
| F6 | 31 | F | 20 | Benign | MEN2/*RET* | Pheochromocytoma |
| F7 | 58 | M | 25 | Benign | PGL1/*SDHD* | Paraganglioma |
| F11 | 23 | M | 40 | Benign | VHL/*VHL* | Pheochromocytoma |
| F24 | 50 | M | 15 | Benign | MEN2/*RET* | Pheochromocytoma |
| F30 | 65 | F | 14 | Benign | MEN2/*RET* | Pheochromocytoma |
| F47 | 24 | F | 45 | Benign | PGL1/*SDHD* | Pheochromocytoma |
| F60 | 46 | F | 11 | Benign | PGL1/*SDHD* | Pheochromocytoma |
| F61 | 26 | F | 55 | Benign | VHL/*VHL* | Pheochromocytoma |

F, Female; M, male.

**Table S3. Germline and somatic genetic variants in previously known pheochromocytoma-associated genes identified with exome sequencing.**

| **Case ID** | **Gene** | **Mutation** | **Germline or somatic** | **Protein alteration** | **PolyPhen-2 prediction (score)(3)** | **Comment** | **Conclusion** |
| --- | --- | --- | --- | --- | --- | --- | --- |
| 32 | *KIF1Bβ* | c.5285G>A | Germline | Arg1762His^a^ | Benign (0.010) |  | Benign variant |
| 61 | *NF1* | c.7642A>G | Germline | Ile2548Val^a^ | Benign (0.008) |  | Benign variant |
| 64 | *SDHD* | c.149A>G | Germline | His50Arg^b^ | Probably damaging (0.985) | Rare polymorphism(4) | Variant of unknown significance |
| 66 | *EGLN2* | c.431C>A | Germline | Ala144Glu^c^ | Benign (0.023) |  | Benign variant |
| 67 | *SDHB* | c.664delT | Germline | Frameshift^a^ | - (Truncating) |  | Damaging mutation |
| 57 | *NF1* | c.4798_ 4799delAA | Somatic | Frameshift^a^ | - (Truncating) |  | Damaging mutation |
| 60 | *RET* | c.2753T>C | Somatic | Met918Thr^d^ | Probably damaging (0.999) | Known activating mutation(5) | Damaging mutation |
| 61 | *NF1* | c.7715delT | Somatic | Frameshift^a^ | - (Truncating) |  | Damaging mutation |
| 62 | *VHL* | c.284C>G | Somatic | Pro95Arg^d^ | Probably damaging (1.000) | Previously observed in pheochromo-cytoma(6) | Damaging mutation |
| 64 | *MAX* | c.328C>T | Somatic | Gln110X^d^ | - (Truncating) |  | Damaging mutation |
| 66 | *NF1* | c.289C>T | Somatic | Gln97X^a^ | - (Truncating) |  | Damaging mutation |
| 68 | *HRAS* | c.37G>C | Somatic | Gly13Arg^d^ | Probably damaging (0.997) | Known activating mutation(7) | Damaging mutation |

^a^Variant previously not reported in Ensembl.

^b^Also reported with a global allele frequency 0.009 in Ensembl, but cannot be excluded as a risk factor due to the predicted damaging effect and a lack of case-control studies.

^c^Reported in Ensembl with allele frequency <0.001.

^d^Variant reported in Ensembl without allele frequency.

**Table S4. Summary of *FGFR1-3* sequence variants and copy number alterations.**

No fusion genes involving *FGFR1* or *FGFR3* were detected.

| **Case ID** | ***FGFR1* copy number** | ***FGFR1-3* sequence variants** | **Relative gene expression** |
| --- | --- | --- | --- |
| 32 | 1.36 |  | 0.0431 |
| 33 | 1.28 |  | No data |
| 34 | 1.23 |  | 0.3050 |
| 35 | 1.92 |  | 0.1040 |
| 36 | 1.91 |  | 0.0767 |
| 37 | 2.00 |  | 0.0724 |
| 38 | 2.05 |  | 0.0433 |
| 40 | 3.86 | ***FGFR1* Asn546Lys^c^** | 0.1580 |
| 41 | 2.08 |  | 0.0481 |
| 42 | 2.03 |  | 0.1190 |
| 44 | 2.02 |  | 0.0492 |
| 45 | 2.03 |  | 0.0556 |
| 46 | 1.94 |  | 0.0603 |
| 47 | 2.21 |  | No data |
| 48 | 1.25 |  | 0.0165 |
| 49 | 1.99 |  | No data |
| 50 | 1.97 | ***FGFR1* Lys656Glu^c^** | 0.0146 |
| 51 | 1.96 |  | 0.0330 |
| 52 | 1.99 |  | 0.05533 |
| 53 | 1.93 |  | No data |
| 54 | 2.03 |  | 0.0629 |
| 57 | 1.93 |  | 0.0701 |
| 58 | 1.12 |  | 0.0360 |
| 60 | 1.97 |  | 0.0406 |
| 61 | 1.84 |  | 0.0260 |
| 62 | 1.92 |  | 0.0315 |
| 63 | 2.02 |  | 0.0457 |
| 64 | 2.09 | ***FGFR1* Asn546Lys^c^** | 0.0452 |
| 66 | 2.06 |  | 0.0196 |
| 67 | 2.00 |  | 0.1200 |
| 68 | 1.98 |  | 0.0721 |
| F1 | 0.93 | *FGFR3* Phe384Leu^d^ | 0.0140 |
| F2^a^ | 2.05 | *FGFR3* Phe384Leu^d^ | 0.0430 |
| F3 | 1.94 |  | 0.0478 |
| F4^a^ | 1.42 |  | 0.0346 |
| F5 | 1.98 |  | 0.0449 |
| F6^a^ | 2.07 |  | 0.0309 |
| F7^a,b^ | 1.95 |  | No data |
| F8 | 1.78 |  | 0.0476 |
| F9 | 2.11 |  | 0.0875 |
| F10 | 1.95 |  | 0.1060 |
| F11^a^ | 2.32 | *FGFR3* Phe384Leu^d^ | 0.0277 |
| F12 | 2.22 |  | 0.1210 |
| F14 | 1.53 |  | 0.0107 |
| F15 | 3.29 |  | 0.7880 |
| F16 | 1.81 |  | 0.0568 |
| F17 | 1.90 |  | 0.0574 |
| F18 | 2.52 |  | 0.0750 |
| F19 | 2.08 |  | 0.0824 |
| F20 | 1.99 |  | 0.0560 |
| F21 | 2.04 |  | No data |
| F22 | No data |  | No data |
| F23 | 1.95 |  | 0.0863 |
| **Case ID** | ***FGFR1* copy number** | ***FGFR1-3* sequence variants** | **Relative gene expression** |
| F24^a^ | 1.41 |  | 0.0160 |
| F25 | 2.06 |  | 0.0610 |
| F26 | 1.33 |  | 0.0825 |
| F27^b^ | 2.05 |  | 0.0430 |
| F28 | 2.04 |  | No data |
| F29 | 1.87 |  | 0.0410 |
| F30^a^ | 1.80 |  | 0.0242 |
| F31 | 2.18 |  | 0.0471 |
| F32 | 2.07 |  | 0.0581 |
| F33 | 1.94 |  | 0.0176 |
| F34 | 1.56 |  | 0.0324 |
| F35 | 1.68 |  | 0.0065 |
| F36 | 1.96 | *FGFR1* Asp127Glu^d^ | 0.2070 |
| F37 | 1.98 |  | 0.0553 |
| F38 | 1.91 |  | 0.0304 |
| F39 | 1.93 |  | 0.0158 |
| F40 | 1.96 |  | 0.0167 |
| F41 | 2.02 |  | 0.0659 |
| F42 | 1.40 |  | 0.1290 |
| F43^b^ | 2.06 |  | 0.0439 |
| F44 | 2.04 |  | 0.0245 |
| F45 | 1.91 |  | 0.0456 |
| F46 | 1.94 |  | 0.0504 |
| F47^a^ | 2.02 |  | 0.0259 |
| F48 | 1.97 |  | 0.0362 |
| F49 | 0.19 |  | 0.1330 |
| F50 | 1.99 |  | 0.0520 |
| F51 | 2.10 |  | 0.1490 |
| F52 | 1.17 |  | 0.0198 |
| F53 | 1.99 |  | 0.0454 |
| F54 | 2.03 |  | 0.0237 |
| F55 | 1.99 |  | 0.0803 |
| F56 | 2.13 |  | 0.0110 |
| F57 | 1.99 |  | 0.0310 |
| F58 | 2.07 |  | 0.0269 |
| F59 | 1.39 |  | 0.0520 |
| F60^a^ | 1.76 |  | 0.0354 |
| F61^a^ | 1.94 |  | 0.1870 |
| F62 | 2.85 |  | 0.2270 |

No data- DNA or RNA unavailable.

^a^Hereditary cases.
^b^Paragangliomas.

^c^Hotspots.

^d^Polymorphisms.

**Table S5. Primers for Sanger sequencing of hotspot regions in *FGFR1*.**

| **Exon^a^** | **Forward primer** | **Reverse primer** | **PCR annealing temperature** | **Hotspot covered** |
| --- | --- | --- | --- | --- |
| *FGFR1* exon 4 | 5’- actaagggagcagtgggact -3’ | 5’- cttcctcccctttcagcctt -3’ | 60°C | Thr141 |
| *FGFR1* exon 12 | 5’- ggatgaagtggggaggagag -3’ | 5’- aagcagcctctcttaacccc -3’ | 55°C | Asn546 |
| *FGFR1* exon 14 | 5’- cagtgtggcagaagttctatga -3’ | 5’- tgaaagcagcacaggggag -3’ | 55°C | Lys656 |

^a^According to Ensembl entry ENST00000447712.

**Table S6. Primers for Sanger sequencing of hotspot regions in *FGFR2* and *FGFR3.***

| **Exon^a^** | **Forward primer** | **Reverse primer** | **PCR annealing temperature** | **Hotspot covered** |
| --- | --- | --- | --- | --- |
| *FGFR2* exon 7 | 5’- ggcttttctggcatgaggtc -3’ | 5’- tcaaagaacctgtggccaaa -3’ | 60°C | Ser252 |
| *FGFR2* exon 9 | 5’- cgtcagtctggtgtgctaac -3’ | 5’- ccagaatcactcgcacatgg -3’ | 60°C | Cys382 |
| *FGFR2* exon 12 | 5’- gtaggcctttgtcccttcct -3’ | 5’- ggaagcccagccatttctaa -3’ | 60°C | Asn549 |
| *FGFR2* exon 14 | 5’- acccggccacactgtattt -3’ | 5’- aatcggggcaggggaatg -3’ | 60°C | Lys659 |
| *FGFR3* exon 6 | 5’- agtggcggtggtggtgagggag -3’ | 5’- tgtgcgtcactgtacaccttgcag -3’ | 58°C | Ser249 |
| *FGFR3* exon 8 | 5’- caacgcccatgtctttgcag -3’ | 5’- cgggaagcgggagatcttg -3’ | 58°C | Tyr373 |
| *FGFR3* exon 13 | 5’- catggagtacttggcctccc -3’ | 5’- actcggtcaaacaaggcct -3’ | 63°C | Lys650 |
| *FGFR3* exon 15 | 5’- caggtgtctgtcctgggagt -3’ | 5’- ttattcgggaacagcctgaag -3’ | 59°C | Gly697 |

^a^According to Ensembl entries ENST00000358487 and ENST00000260795, respectively.

**Table S7. Primers for Sanger sequencing of hotspot regions in *HRAS*.**

| **Exon^a^** | **Forward primer** | **Reverse primer** | **PCR annealing temperature** | **Hotspot covered** |
| --- | --- | --- | --- | --- |
| *HRAS* exon 2 | 5’- gtgggtttgcccttcagat -3’ | 5’- cgccaggctcacctctat -3’ | 64°C | Gly13 |
| *HRAS* exon 3 | 5’- ggagaggctggctgtgtgaa -3’ | 5’- aaaagacttggtgttgttga -3’ | 61°C | Gln61 |

^a^According to Ensembl entry ENST00000311189.

**Table S8. *HRAS* mutations identified in the cohort.**

| **Case ID** | **Mutation^a^** | **Protein alteration** | **Present in normal tissue** | **Analysis approach** |
| --- | --- | --- | --- | --- |
| 37 | c.182A>G | Gln61Arg | No | Targeted sequencing |
| 41 | c.37G>C | Gly13Arg | No | Targeted sequencing |
| 46 | c.182A>G | Gln61Arg | No | Targeted sequencing |
| 68 | c.37G>C | Gly13Arg | No | Whole-exome sequencing |

^a^According to Ensembl entry ENST00000311189.

**Figure S1.** **Mutation hotspot sites in *FGFR1*, *FGFR2* and *FGFR3* analyzed with Sanger sequencing in this study.**
The mutation statistics histograms were derived from the COSMIC database [26]. Interestingly, the three genes show both unique and homologous hotspots. In addition to analysis of exons containing hotspot sites, the whole length of the genes was investigated in 16 samples analyzed with exome sequencing. *FGFR4* was not investigated in this study (except for samples analyzed with exome sequencing), since only few mutations and no clear hotspots have been reported for this gene.

**
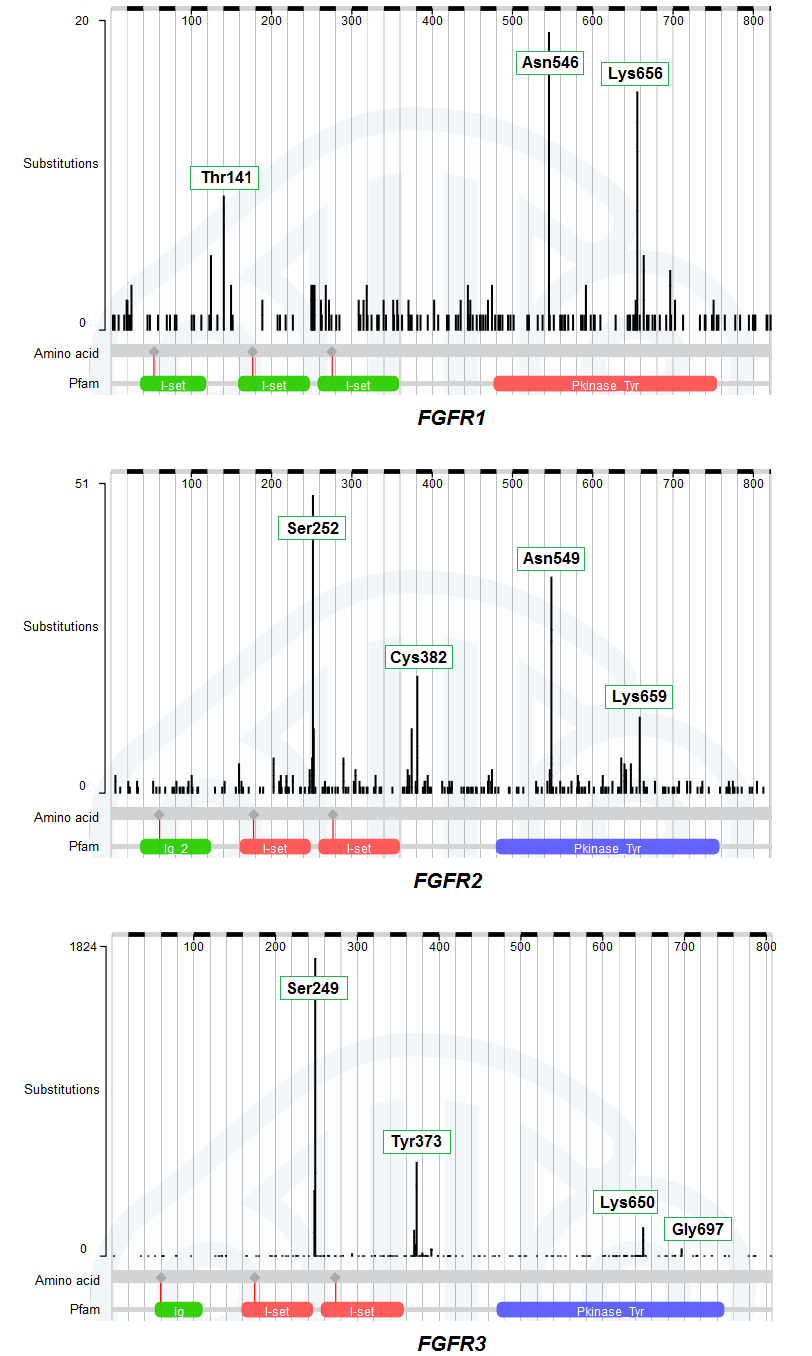
**

**Figure S2. Summary of mutations detected in 31 non-familial pheochromocytomas from Scandinavia.**

Mutations in the known susceptibility genes were mutually exclusive. For the novel gene *FGFR1*, two mutations were detected in tumors without other known mutations, whereas one occurred in combination with a somatic *MAX* mutation. The cases were investigated with whole-exome sequencing in this study and/or previously tested with targeted sequencing (details of the cohort are given in Table S1).


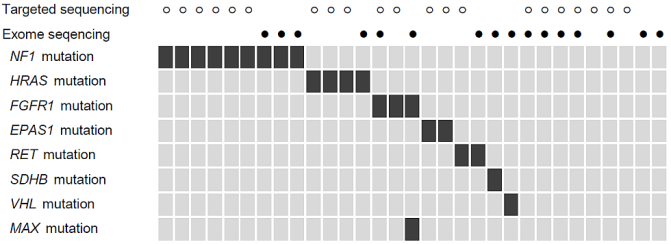


**Figure S3. Example of *FGFR1* copy number data retrieved by digital droplet PCR.**

Case numbers are shown at the bottom and the copy number scale on the left. Copy number values are shown with error bars as 95% confidence intervals. In the tumor DNA of cases 41-47, no copy number alterations were observed. Case 40 had an amplification (3.86 copies) and case 48 was accounted as a deletion (1.25 copies). Data was exported from the QuantaSoft software (BioRad).

**
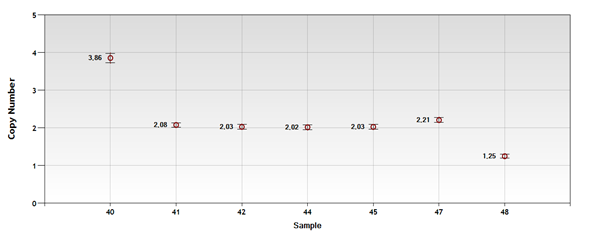
**

**Figure S4. Microarray copy number data from the *FGFR1* locus.**

The figure shows amplification (three copies) in the tumor DNA of case 40. The amplification covered a larger region of about 2.5 Mb on chromosome 8p. The view was exported from the Affymetrix Genotyping Console Browser software. Positions of SNP markers and their copy number are indicated with black dots, and the copy number scale is shown to the left. The bottom panels show genes and base pair positions according to UCSC hg18.

**
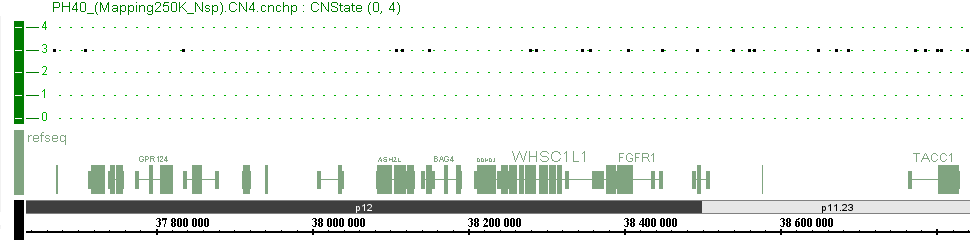
**

**Figure S5. mRNA expression of *FGFR1* in sporadic pheochromocytomas measured by digital droplet PCR analysis.**

Two-tailed Student’s t-tests were used to compare gene expression levels. Data was visualized with GraphPad Prism v.6.03 and horizontal bars represent mean values for the groups. Hereditary cases and paragangliomas were excluded from the statistical analysis. (A) There was a significant difference in expression between tumors with amplified *FGFR1* (n=4) and the group with 2 copies of the gene (n=59). (B) No significant difference in expression was observed between the cases with deletions (n=10) and cases accounted as normal (n=59).


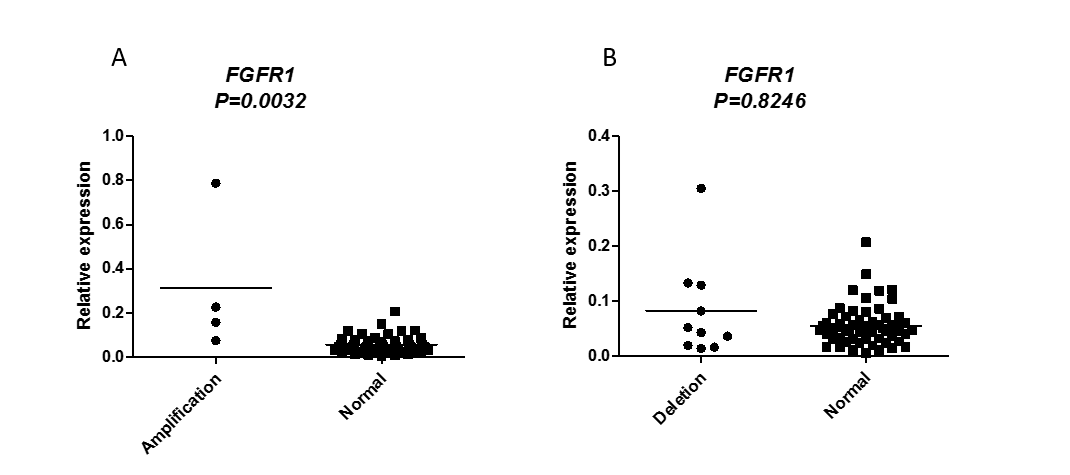


**Supplementary References**

1. Welander J, Andreasson A, Brauckhoff M, et al. (2014) Frequent EPAS1/HIF2alpha exons 9 and 12 mutations in non-familial pheochromocytoma. Endocr Relat Cancer 21: 495-504

2. Li H, Durbin R (2009) Fast and accurate short read alignment with Burrows-Wheeler transform. Bioinformatics 25: 1754-1760

3. McKenna A, Hanna M, Banks E, et al. (2010) The Genome Analysis Toolkit: a MapReduce framework for analyzing next-generation DNA sequencing data. Genome Res 20: 1297-1303

4. DePristo MA, Banks E, Poplin R, et al. (2011) A framework for variation discovery and genotyping using next-generation DNA sequencing data. Nat Genet 43: 491-498

5. Cibulskis K, Lawrence MS, Carter SL, et al. (2013) Sensitive detection of somatic point mutations in impure and heterogeneous cancer samples. Nat Biotechnol 31: 213-219

6. Koboldt DC, Zhang Q, Larson DE, et al. (2012) VarScan 2: somatic mutation and copy number alteration discovery in cancer by exome sequencing. Genome Res 22: 568-576

7. Li H, Handsaker B, Wysoker A, et al. (2009) The Sequence Alignment/Map format and SAMtools. Bioinformatics 25: 2078-2079

8. Cingolani P, Platts A, Wang le L, et al. (2012) A program for annotating and predicting the effects of single nucleotide polymorphisms, SnpEff: SNPs in the genome of Drosophila melanogaster strain w1118; iso-2; iso-3. Fly (Austin) 6: 80-92

9. Welander J, Soderkvist P, Gimm O (2011) Genetics and clinical characteristics of hereditary pheochromocytomas and paragangliomas. Endocr Relat Cancer 18: R253-276

10. Lorenzo FR, Yang C, Ng Tang Fui M, et al. (2013) A novel EPAS1/HIF2A germline mutation in a congenital polycythemia with paraganglioma. J Mol Med (Berl) 91: 507-512

11. Letouze E, Martinelli C, Loriot C, et al. (2013) SDH mutations establish a hypermethylator phenotype in paraganglioma. Cancer Cell 23: 739-752

12. Castro-Vega LJ, Buffet A, De Cubas AA, et al. (2014) Germline mutations in FH confer predisposition to malignant pheochromocytomas and paragangliomas. Human Molecular Genetics 23: 2440-2446

13. Crona J, Delgado Verdugo A, Maharjan R, et al. (2013) Somatic Mutations in H-RAS in Sporadic Pheochromocytoma and Paraganglioma Identified by Exome Sequencing. J Clin Endocrinol Metab 98: E1266-1271

14. Yang C, Zhuang Z, Fliedner SM, et al. (2015) Germ-line PHD1 and PHD2 mutations detected in patients with pheochromocytoma/paraganglioma-polycythemia. J Mol Med (Berl) 93: 93-104

15. Gaal J, Burnichon N, Korpershoek E, et al. (2010) Isocitrate dehydrogenase mutations are rare in pheochromocytomas and paragangliomas. J Clin Endocrinol Metab 95: 1274-1278

16. Wadt K, Choi J, Chung JY, et al. (2012) A cryptic BAP1 splice mutation in a family with uveal and cutaneous melanoma, and paraganglioma. Pigment Cell Melanoma Res 25: 815-818

17. Robinson JT, Thorvaldsdottir H, Winckler W, et al. (2011) Integrative genomics viewer. Nat Biotechnol 29: 24-26

18. Thorvaldsdottir H, Robinson JT, Mesirov JP (2013) Integrative Genomics Viewer (IGV): high-performance genomics data visualization and exploration. Brief Bioinform 14: 178-192

19. Welander J, Larsson C, Backdahl M, et al. (2012) Integrative genomics reveals frequent somatic NF1 mutations in sporadic pheochromocytomas. Human Molecular Genetics 21: 5406-5416

20. Hindson BJ, Ness KD, Masquelier DA, et al. (2011) High-throughput droplet digital PCR system for absolute quantitation of DNA copy number. Anal Chem 83: 8604-8610

21. Irizarry RA, Hobbs B, Collin F, et al. (2003) Exploration, normalization, and summaries of high density oligonucleotide array probe level data. Biostatistics 4: 249-264

22. Benjamini Y, Hochberg Y (1995) Controlling the False Discovery Rate - a Practical and Powerful Approach to Multiple Testing. Journal of the Royal Statistical Society Series B-Methodological 57: 289-300

23. Burnichon N, Vescovo L, Amar L, et al. (2011) Integrative genomic analysis reveals somatic mutations in pheochromocytoma and paraganglioma. Human Molecular Genetics 20: 3974-3985

24. Singh D, Chan JM, Zoppoli P, et al. (2012) Transforming fusions of FGFR and TACC genes in human glioblastoma. Science 337: 1231-1235

25. Di Stefano AL, Fucci A, Frattini V, et al. (2015) Detection, Characterization, and Inhibition of FGFR-TACC Fusions in IDH Wild-type Glioma. Clin Cancer Res 21: 3307-3317

26. Forbes SA, Bindal N, Bamford S, et al. (2011) COSMIC: mining complete cancer genomes in the Catalogue of Somatic Mutations in Cancer. Nucleic Acids Res 39: D945-950
